# Coal and gas protrusion risk evaluation based on cloud model and improved combination of assignment

**DOI:** 10.1038/s41598-024-55382-1

**Published:** 2024-02-24

**Authors:** Yun Qi, Kailong Xue, Wei Wang, Xinchao Cui, Ran Liang, Zewei Wu

**Affiliations:** 1 College of Mechanical Engineering and Automation, Liaoning University of Technology, Jinzhou, 121001 People’s Republic of China; 2 School of Coal Engineering, Shanxi Datong University, Datong, 037000 Shanxi People’s Republic of China; 3 China Safety Science Journal Editorial Department, China Occupational Safety and Health Association, Beijing, 100011 People’s Republic of China

**Keywords:** Coal and gas prominence, Prominence hazard evaluation, Combined assignment, Cloud model, Improved hierarchical analysis (IAHP), CRITIC, Coal, Natural gas, Applied mathematics

## Abstract

The proposed study presents an enhanced combination weighting cloud model for accurate assessment of coal and gas outburst risks. Firstly, a comprehensive evaluation index system for coal and gas outburst risks is established, consisting of primary indicators such as coal rock properties and secondary indicators including 13 factors. Secondly, the improved Analytic Hierarchy Process (IAHP) based on the 3-scale method and the improved CRITIC based on indicator correlation weight determination method are employed to determine subjective and objective weights of evaluation indicators respectively. Additionally, the Lagrange multiplier method is introduced to fuse these weights in order to obtain optimal weights. Subsequently, a prominent danger assessment model is developed based on cloud theory. Finally, using a mine in Hebei Province as an example, the results obtained from IAHP combined with improved CRITIC weighting method are compared with those from traditional AHP method and AHP-CRITIC combination weighting method. The findings demonstrate that among all methods considered, IAHP combined with improved CRITIC exhibits superior performance in terms of distribution expectation Ex, entropy value En, and super entropy He within cloud digital features; thus indicating that the risk level of coal and gas outbursts in this particular mine can be classified as general risk. These evaluation results align well with actual observations thereby validating the effectiveness of this approach. Consequently, this constructed model enables rapid yet accurate determination of coal and gas outburst risks within mines.

## Introduction

In 2022, China's total energy consumption will be 5.41 billion tons of standard coal, representing 56.2 percent of total energy consumption. In recent years, China's coal mines have been continuously expanding into deeper seams at a rate of 0.67–1 m per month^1^. At present, there are about 47 mines covering more than one kilometer, of which 1218 have experienced outbursts. As the depth of coal mining continues to deepen, the frequency, scope and intensity of prominent disasters continue to increase, causing significant losses to the lives and property of production personnel. To this day, coal and gas outburst accidents have become one of the main factors affecting coal mine safety production^2,3^. Therefore, in order to ensure mine production and personnel safety, predicting the risk of coal and gas outbursts in mines is fundamental and key to ensuring safe mine production^4^.

The risk assessment of coal and gas outbursts has been extensively studied by domestic and international scholars, and many different evaluation methods and models have been proposed. For example, Yun^5^ used the comprehensive evaluation method constructed by the improved fuzzy analytic hierarchy process (IFAHP) and the entropy weight method with dynamic adjustment (EWMDA) to evaluate the risk of coal and gas outburst in the Pingdingshan mining area, achieving rapid prediction of regional coal and gas outburst risks, and the evaluation results are essentially consistent with the actual outburst risk situation. Xionggang et al.^6^ used a combination of entropy weight method and extension theory to construct a coal and gas outburst evaluation model. The burst risk level obtained in the actual evaluation of the model is consistent with the actual situation of the mine. Bing et al.^7^ proposed an intelligent weighted grey target decision-making model for outburst risk assessment based on grey system theory, which improved the scientificity and accuracy of outburst prediction. Yongming et al.^8^ proposed a risk assessment model for coal and gas outburst in the 11,041 working face of Fangshan Mine based on fuzzy comprehensive evaluation, which verified the rationality of the model. Chen Liuyu et al.^9^ constructed an AHP-TOPSIS impact coal and gas outburst tendency evaluation model, and verified the rationality of the model through on-site prediction of Fangshan Mine in Pingyu Mining Area. Yu Liya et al.^10^ established a comprehensive evaluation model for the risk of coal and gas outburst based on cloud models and D-S theory. Evaluation results in emerging coal mines have demonstrated the high accuracy of the model. Cai Junjie et al.^11^ obtained more accurate and reliable objective evaluation weights by combining the advantages of entropy weight theory and attribute mathematics theory. Xu Enyu et al.^12^ first proposed the AHP-GT model for evaluating coal and gas outburst, which was applied in coal mines in Guizhou Province, and the evaluation results were consistent with the actual measurement. Ding Haojiang^13^ established an unascertained theoretical measurement model to balance the determination and uncertainty of evaluation indicators, and conducted coal and gas outburst risk assessment in Xinxing Coal Mine. The results show that the model has a high evaluation accuracy. Tang Meng et al.^14^ constructed a coal and gas outburst risk assessment model based on game theory and TOPSIS method. The model considers factors comprehensively and avoids the one-sidedness of individual indicators. The on-site evaluation results of a certain mine in Tongzi County are consistent with the actual measurements, validating its reliability. Haifei et al.^15^ constructed a dynamic prediction model for coal and gas outburst based on multiple algorithms and multivariate analysis, identified 8 optimal classification models, and conducted grade prediction on 8 typical coal and gas outburst accident cases. The results show that the constructed multi-parameter, multi-algorithm, multi-group and multi-judgment index collaborative prediction models for coal and gas outbursts have high accuracy and some generality, which provides a new approach for predicting the risk level of coal and gas outbursts. Quanjie et al.^16^ constructed an outburst risk assessment model based on entropy weight method and grey target theory. By analyzing the outbursts in multiple coal mines in Guizhou, we have demonstrated that the model can accurately assess the risk of outbursts, which validates the rationality and feasibility of the model. Although the aforementioned studies have achieved some results in assessing the risk of coal and gas outbursts, the evaluation results are not reliable due to the incompleteness of the factors affecting the risk indicators of coal and gas outbursts, and the ambiguous relationship between primary and secondary factors. The rationality of subjective and objective weight distributions has a significant impact on the evaluation results, and the uncertainty, ambiguity and discreteness of the metrics cannot be taken into account, resulting in a lack of reliability in the evaluation results.

In view of this, the author uses the Improved Analytic Hierarchy Process (IAHP) and the improved Criterion Importance Through Correlation (CRITIC) to determine the subjective and objective weights of evaluation indicators, and introduces cloud theory to construct a cloud model for evaluating the risk of coal and gas outburst based on combination weighting. By comparing and analyzing the cloud distribution characteristics of comprehensive evaluation and standard evaluation, the evaluation level of outburst danger is determined. Taking a certain mine in Hebei as an example, the rationality of the model is verified, in order to provide a theoretical basis for the prevention and control of coal and gas outburst hazards.

## Construction of the appraisal index system

The comprehensive action hypothesis is widely recognized in the mechanism of coal and gas outbursts, positing that geostress, gas composition, and coal rock properties exert primary influences on such occurrences^3^.

1. Factors related to coal rock properties determine the occurrence and development difficulty of outbursts, which are influenced by factors such as coal's firmness coefficient, type of coal damage, thickness of the coal seam, and dip angle of the coal seam. The risk of outburst increases with a smaller firmness coefficient and softer coal body^17^. According to "Identification Specification for Coal and Gas Outburst Mines" (AQ 1024–2006), types of coal damage are classified into five categories: Class 1 represents non-destructive coal, Class 2 represents destructive coal, Class 3 represents strongly destructive coal, Class 4 represents crushed coal, and Class 5 represents fully powdered coal. A thicker coal seam indicates poorer stability and higher likelihood of causing an outburst. Additionally, a larger dip angle amplifies the impact from self-weight on the stability of the seam and increases the risk of outbursts.
2. Gas factors play a crucial role in the occurrence and propagation of coal and gas outbursts, primarily by facilitating the ejection and transportation of coal during such events^18^. When underground gas pressure and gas content reach an extreme threshold, the likelihood of outburst significantly increases. Moreover, higher initial velocity of gas release leads to faster desorption and release of gas from the coal body, thereby amplifying the potential for gas outbursts.
3. Geological factors: Ground stress is the primary factor contributing to protrusions, encompassing rock stress, concentrated stress, and tectonic stress^19^. The burial depth plays a pivotal role in determining the stress and concentration of rock layers. Increasing the burial depth of coal seams promotes gas occurrence, thereby elevating the likelihood of coal and gas outbursts. Structural stress arises from geological tectonic processes and correlates with factors such as structural complexity. Based on characteristics like faults, folds, and joints, structures can be categorized into four levels of complexity: 1 represents simple structures; 2 represents general structures; 3 represents relatively complex structures; and 4 represents extremely complex structures^20^.

**Figure 1:**
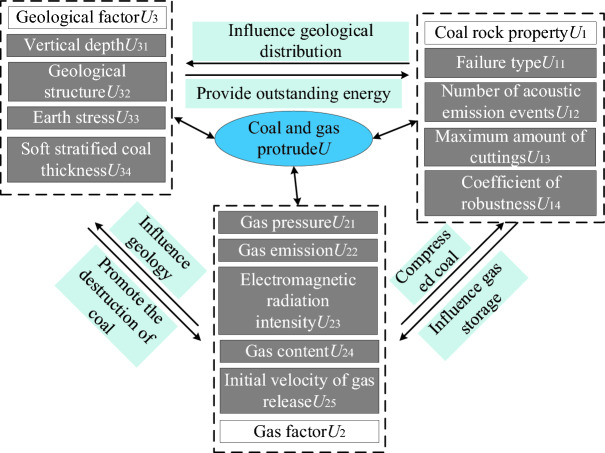
Coal and gas outstanding risk evaluation index system.

Based on the aforementioned analysis, a set of 13 key factors were selected as predictive indicators for coal and gas outbursts. These factors include coal failure type (*U*_11_), number of acoustic emission events (*U*_12_), maximum drilling debris amount (*U*_13_), solidity coefficient (*U*_14_), gas content (*U*_21_), gas emission amount (*U*_22_), electromagnetic radiation intensity (*U*_23_), gas pressure (*U*_24_), initial gas release velocity (*U*_25_), vertical depth (*U*_31_), geological structure(*U*_32_), geostress(*U*_33_), and soft layered coal thickness(*U*_34_). The establishment of an evaluation system for the aforementioned influencing factors is depicted in Fig. 1.

## Construct a modified combinatorial weight cloud model to evaluate the model

### Combination weighting method

Combination weighting is a comprehensive weight weighting algorithm that combines multiple subjective and objective weight calculation methods based on the advantages of different weight calculation methods, utilizing certain mathematical relationships to obtain the optimal combination weight and eliminate the influence of subjective and objective factors on indicator weigh.

#### Improving the analytic hierarchical process to determine subjective weights

The Analytic Hierarchy Process (AHP) method is a subjective weighting approach based on expert scoring, which is currently widely employed in risk assessment tasks. However, the traditional AHP method often faces challenges as the judgment matrix fails to meet the consistency test. Consequently, it necessitates multiple reconstructions of the judgment matrix until passing the consistency test, resulting in a significant increase in workload without practical significance^21^. To address this issue, an improved AHP method (IAHP) utilizes the scaling construction technique for constructing a judgment matrix. It determines scaling values between adjacent factors through factor importance ranking and subsequently employs these values to determine other elements within the matrix. As a result, regardless of scaling choice, the constructed matrix maintains consistency^22^. In this article, we adopt the 3-scale method to establish a comparison matrix while simultaneously utilizing exponential functions for constructing an optimal transfer matrix. This enhances consistency analysis of the judgment matrix by simplifying calculations and making them more reasonable while reducing subjective influences on weights. The specific calculation steps are as follows:

1. The initial comparison matrix is constructed to accurately depict the relative importance relationship among different indicators within the same constraint. To achieve this, the 3-scale method is employed for comparing indicator importance under various constraint conditions, resulting in a comparison matrix *A* = (*a*_*ij*_) _n × n_. Here, *A*_*ij*_ represents the significance of factor *i* compared to factor* j* in matrix *A*, where *i* and *j* range from 1 to n, satisfying *a*_*ii*_ = 0 and* a*_*ij*_ + *a*_*ji*_ = 0. The specific meanings associated with different values of *a*_*ij*_ are presented in Table 1.
2. The establishment of the optimal judgment matrix involves utilizing the linear weighting method to perform a linear transformation on each element in *A*, aiming to reduce human error and minimize the influence of inferior elements in the anti-symmetric matrix on evaluation results. Additionally, an exponential function is introduced to optimize the transformed elements, thereby obtaining the most favorable judgment matrix *R*.
  1 $$r_{ij} = \frac{1}{n}\sum\limits_{i = 1}^{n} {(a_{in} + a_{nj} )}$$
  2 $$d_{in} = \exp (r_{ij} )$$
  3 $${\varvec{R}} = \left( {\begin{array}{*{20}c} {d_{11} } & {d_{12} } & \cdots & {d_{1n} } \\ {d_{21} } & {d_{22} } & \cdots & {d_{2n} } \\ \vdots & \vdots & \vdots & \vdots \\ {d_{n1} } & {d_{n2} } & \cdots & {d_{nn} } \\ \end{array} } \right)$$
3. The International Analytic Hierarchy Process (IAHP) establishes subjective weights. To streamline the calculation process, the widely adopted approach is to employ the root method for obtaining subjective weighted weight. The calculation method for this approach is as follows:
  4 $$\overline{{w_{{{\text{A}}i}} }} = \left( {\mathop \prod \limits_{i = 1}^{n} d_{in} } \right)^{1/n} /\sum\limits_{i = 1}^{n} {\left( {\mathop \prod \limits_{i = 1}^{n} d_{in} } \right)^{1/n} }$$
  5 $$w_{{{\text{A}}i}} { = }\overline{{w_{{{\text{A}}i}} }} /\sum\limits_{i = 1}^{n} {\overline{{w_{{{\text{A}}i}} }} }$$
  6 $$w_{{\text{A}}} = (w_{{{\text{A}}1}} ,w_{{{\text{A}}2}} , \ldots ,w_{{{\text{A}}n}} )^{T}$$

**Table 1:** Meaning of *a*_*ij*_ scale value.

*a*_*ij*_ scale value	1	0	−1
implication	Index *i* is more important than *j*	Indicator *i* is as important as indicator *j*	Index *i* is less important than *j*

#### Determination of objective weights based on the modified CRITIC method

The CRITIC weighting method is an objective approach based on the volatility and conflict of data. Volatility is measured by standard deviation, with higher weights assigned to indicators exhibiting greater volatility. Conflict is assessed using correlation coefficients, where lower weights are given to indicators with stronger correlations. The final weight is obtained by multiplying the contrast intensity with the conflict indicator and normalizing it^23,24^. Although the CRITIC method effectively considers data conflict and volatility, it does not account for the degree of dispersion between indicator data. Therefore, we propose incorporating the entropy principle to enhance the CRITIC method, enabling it to comprehensively consider three key attributes: correlation, contrast strength, and dispersion of indicator data. Entropy serves as a measure of uncertain information that is inversely proportional to its quantity; thus smaller entropy values correspond to higher weights. The specific steps for improvement are as follows:

1. The proportion of indicator values Pij should be calculated for each scheme and under each indicator.
  7 $$\left\{ {\begin{array}{*{20}c} {P_{ij} = \frac{{x_{ij}^{\prime } }}{{\sum\limits_{i = 1}^{n} {x_{ij}^{\prime } } }}} \\ {\sum {P_{ij} } = 1} \\ \end{array} } \right.$$
  In Equation,* x*_*ij*_' represents the standardized data.
2. Calculate the information entropy *e*_*j*_ of the index.
  8 $$\left\{ {\begin{array}{*{20}l} {e_{j} = - \xi \sum\limits_{i = 1}^{m} {P_{ij} } \cdot \ln P_{ij} } \hfill \\ {\xi = \frac{1}{\ln n}} \hfill \\ \end{array} } \right.$$
  In Equation, *ξ* ensure that the information entropy is meaningful.
3. At this time, the calculation formula of weight *W*_*j*_ of the improved CRITIC method can be obtained.
  9 $$W_{j} = \frac{{(e_{j} + S_{j} )\sum\limits_{i = 1}^{n} {(1 - \left| {l_{ij} } \right|)} }}{{\sum\limits_{j = 1}^{m} {(e_{j} + S_{j} )\sum\limits_{i = 1}^{n} {(1 - \left| {l_{ij} } \right|)} } }}$$

In Equation, *l*_*ij*_ represents the row* i* column *j* element in the conflict matrix^25^.

#### Combine weight

In order to accurately reflect the risk assessment capabilities of coal and gas outbursts, the decision-maker needs to integrate subjective and objective weights for the weighting calculation. Therefore, the Lagrange multiplier method is used to calculate the integrated weights of the various metrics, namely* q*_*j*_:

10 $$q_{j} = \frac{{\sqrt {w_{Ai} W}_{j} }}{{\sum\limits_{i = 1}^{m} {\sum\limits_{j = 1}^{n} {\sqrt {w_{Ai} W}_{j} } } }}$$

11 $$Q = (q_{1} ,q_{2} ,q_{3} , \cdots ,q_{j} )$$

In Equation, *Q* is the optimal integrated weight vector and the optimal weight for each indicator is *q*_*i*_.

### Construct a comprehensive evaluation model

**Figure 2:**
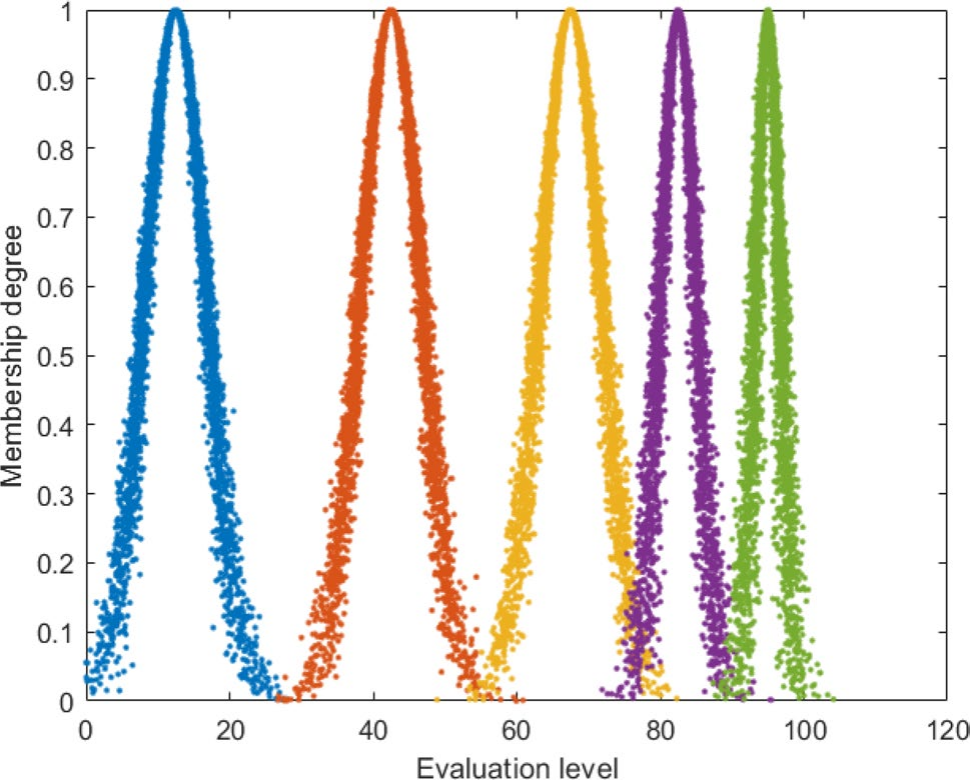
Standard evaluation cloud map.

The cloud model was first proposed by Professor Li in 1995^26^. This model is a model that leverages methods such as membership clouds, digital features, and cloud generators to transform uncertainty into a qualitative and quantitative description. Currently, this approach has been widely used in disaster assessment, emergency path optimization, and other areas. The standard evaluation cloud is shown in Fig. 2.Specific measures for implementation are as follows:

1. Determination of cloud feature values. The risk assessment of coal and gas outburst can be divided into 5 levels in order, namely I (safe), II (relatively safe), III (general), IV (relatively dangerous), and V (dangerous), The cloud eigenvalue, (*E*_*xk*_, *E*_*nk*_, *H*_*ek*_), is computed as follows:
  12 $$\left\{ {\begin{array}{*{20}c} {Exk = (c_{k}^{\max } + c_{k}^{\min } )/2} \\ {Enk = (c_{k}^{\max } - c_{k}^{\min } )/6} \\ {Hek = k} \\ \end{array} } \right.$$
  In Equation, $c_{k}^{\min }$ is the minimum value of the expert's score for each indicator; $c_{k}^{\max }$ is the maximum score given by the expert for each metric; Also, *E*_*xk*_ stands for expectation; *E*_*nk*_ represents entropy; *H*_*ek*_ represents hyperentropy; *k* is usually a constant with a value of 0.5.
  The method for calculating cloud models is:
  1. Randomly generate a set of numbers $E_{n}^{\prime }$, which conform to a normal distribution with expectation *H*_*e*_, and variance *E*_*n*_.
  2. Generate a 1-cloud droplet consisting of a normal random number *x*_*i*_ with expectation value *E*_*x*_, and variance $E_{n}^{\prime_2}$.
  3. To obtain the membership *γ*_*i*_ of the cloud model, use Eq. (21) for calculation.
    13 $$\gamma_{i} = \exp \left( {\frac{{(x - E_{x} )^{2} }}{{2x_{i}^{2} }}} \right)$$
  In Equation, *x* denotes the score of each sample indicator.
  According to references^10,22^, the range of coal and gas outburst risk level scores is divided, and the standard cloud model parameters are obtained according to Eq. (21), as shown in Table 2.

**Table 2:** Standard cloud model parameters.

Outstanding hazard level	Score range	Cloud model parameters
V	[90.0,100]	(95.0,1.7,0.5)
IV	[75.0,90.0)	(82.5,2.5,0.5)
III	[60.0,75.0)	(67.5,4.2,0.5)
II	[25.0,60.0)	(42.5,4.2,0.5)
I	[0.0,25.0)	(12.5,4.2,0.5)
2. Determine the evaluation indicator cloud. (*E*_*xj*_, *E*_*nj*_, *H*_*ej*_) is calculated based on the coal and natural gas outbursts risk index data required to compute the evaluation index cloud, which is calculated as follows:
  14 $$\left\{ {\begin{array}{*{20}c} {E_{xj} = \frac{1}{n}\sum\limits_{i = 1}^{n} {Z_{i} } } \\ {E_{nj} = \sqrt {\frac{{\uppi }}{2}} \cdot \frac{1}{n}\sum\limits_{i = 1}^{n} {\left| {Zi - \overline{Z} } \right|} } \\ {S_{j}^{2} { = }\frac{1}{n - 1}\sum\limits_{i = 1}^{n} {(Z_{j} - \overline{Z} )^{2} } } \\ {H_{ej} = \sqrt {\left| {S_{j}^{2} - E_{nj}^{2} } \right|} } \\ \end{array} } \right.$$
  In Equation, $S_{j}^{2}$ denotes the variance of the sampled data; *H*_*ej*_ denotes the superentropy of the sampled data; *E *_*nj*_ denotes the entropy of the sampled data; *E*_*xj*_ denotes the expectation of the sampled data; $\overline{Z}$ and Z_*i*_ denote the mean and numerical value of the sampled data, respectively; *n* denotes the number of samples taken.
3. Generate a comprehensive evaluation cloud. The integrated IAHP method and the modified CRITIC method are applied to the cloud model to obtain the integrated weight values. The integrated evaluation cloud is generated by a reverse cloud generator, and the distribution characteristics of the integrated evaluation cloud and the standard evaluation cloud are compared to obtain the final evaluation result, which determines the risk assessment level for coal and gas outbursts.
  15 $$\left\{ {\begin{array}{*{20}c} {E_{x} = \sum\limits_{i = 1}^{n} {(E_{xj} \cdot q_{j} )} } \\ {E_{n} = \sqrt {\sum\limits_{j = 1}^{n} {(E_{nj}^{2} \cdot q_{j} )} } } \\ {H_{e} = \left( {\sum\limits_{j = 1}^{n} {(H_{ej} \cdot q_{j}^{2} )} } \right)/\sum\limits_{j = 1}^{n} {q_{j}^{2} } } \\ \end{array} } \right.$$

In Equation, *E*_*x*_, *E*_*n*_, and *H*_*e*_ are the expected entropy and superentropy of the integrated evaluation cloud, respectively.

**Figure 3:**
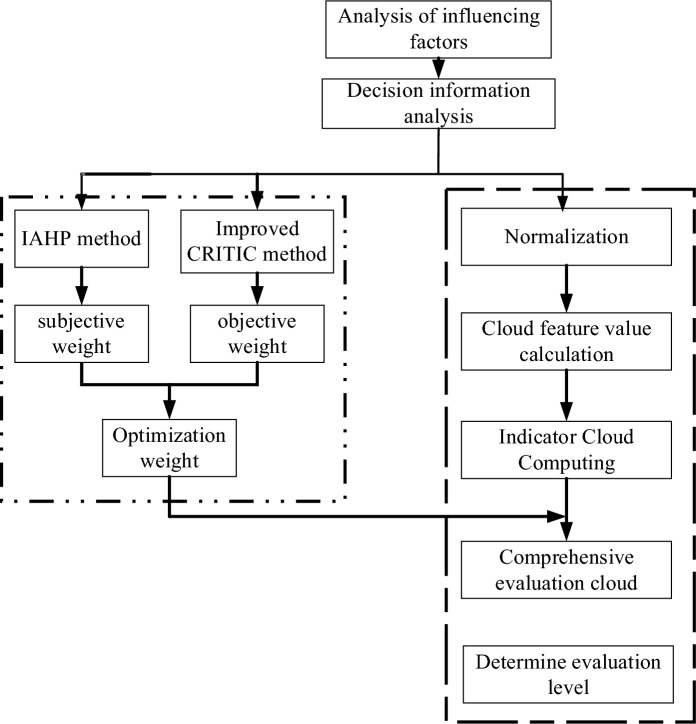
Flow chart for coal and gas protrusion risk assessment.

A diagram of the risk assessment process for coal and gas outbursts is shown in Fig. 3. The steps are as follows: First, the evaluation objective is analyzed and an evaluation metric is selected. Based on this, the subjective and objective weights of each metric are determined using the IAHP method and the modified CRITIC method, which are coupled to obtain the optimal weights. Then, they are multiplied with the eigenvalues of the cloud to obtain a comprehensive evaluation of the cloud. Finally, by overlaying the standard cloud with the obtained synthetic evaluation cloud, a synthetic evaluation cloud map can be generated and the evaluation results can be obtained by observations.

## Engineering case application

### Data acquisition

In the case of a mine in Hebei province, 10 experts in the field of mining engineering and safety assessment were hired to qualitatively assess the risk of coal and gas explosion using a modified comprehensive assessment model. The thickness of the coal bearing strata in this mining area is 600–1,050 m, with an average depth of 729 m and an average strike length of 173 m. The average dip angle of the coal seam is 8°, and the average thickness of the coal seam is 3.5 m. The geology of the face of the works is relatively complex, the roof is poorly stable, the pressure is high, and it is liable to break and fall off, so there is a hidden danger of coal and gas explosions.

### Determining indicator weights

#### Establishing subjective weights using the IAHP method

We will recruit 10 experts specialized in mining engineering and safety evaluation to employ an enhanced comprehensive evaluation model for qualitative assessment of coal and gas outburst risks. To ensure the accuracy and effectiveness of the risk assessment results, as well as minimize subjective influences, all invited experts are scientific researchers with ample field experience. Based on the coal and gas outburst risk evaluation index system established in this article, a comparison matrix *A*_0_ to *A*_3_ is constructed for all evaluation indicators using step 1) from Chapter 2.2.1 along with expert scoring outcomes.

$${\varvec{A}}_{{0}} { = }\left( {\begin{array}{*{20}c} 0 & { - 1} & { - 1} \\ 1 & 0 & { - 1} \\ 1 & 1 & 0 \\ \end{array} } \right)\quad {\varvec{A}}_{{1}} { = }\left( {\begin{array}{*{20}c} 0 & { - 1} & { - 1} & 0 \\ 1 & 0 & 1 & { - 1} \\ 1 & { - 1} & 0 & { - 1} \\ 0 & 1 & 1 & 0 \\ \end{array} } \right)\quad {\varvec{A}}_{2} { = }\left( {\begin{array}{*{20}c} 0 & { - 1} & { - 1} & { - 1} & { - 1} \\ 1 & 0 & 1 & { - 1} & { - 1} \\ 1 & { - 1} & 0 & { - 1} & { - 1} \\ 1 & 1 & 1 & 0 & 1 \\ 1 & 1 & 1 & { - 1} & 0 \\ \end{array} } \right),\quad {\varvec{A}}_{3} { = }\left( {\begin{array}{*{20}c} 0 & { - 1} & 1 & 1 \\ 1 & 0 & 1 & 1 \\ { - 1} & { - 1} & 0 & 1 \\ { - 1} & { - 1} & { - 1} & 0 \\ \end{array} } \right)$$

The subjective weights of IAHP, calculated using Eqs. (1)–(5), are as follows: *w*_*A*0_ = (0.148,0.289,0.563); *w*_*A*1_ = (0.141,0.297,0.180,0.382);*w*_*A*2_ = (0.077,0.381,0.115, 0.171,0.256);*w*_*A*3_ = (0.292,0.481,0.176,0.051). The weights of each index obtained through the IAHP method are as follow: *W*_*A*_ = (0.021, 0.044, 0.027, 0.057, 0.022, 0.110, 0.035, 0.049, 0.074, 0.164, 0.272, 0.099, 0.026).

#### Improving the CRITIC method for establishing objective weights

In order to validate the accuracy and rationality of the enhanced combination weighting cloud model for coal and gas outburst risk, a total of 20 sets of historical outburst data were collected from a mine located in Hebei Province. The improved CRITIC method, incorporating fusion Eqs. (7) to (9), was employed to calculate and analyze the coal and gas outburst risk at another mine in Hebei Province. The specific data is presented in Table 3.

**Table 3:** Highlighting data sets and evaluation index systems^27^.

Number	*U*_11_	*U*_12_	*U*_13_	*U*_14_	*U*_21_	*U*_22_	*U*_23_	*U*_24_	*U*_25_	*U*_31_	*U*_32_	*U*_33_	*U*_34_
1	3	885	8.88	0.58	10.24	6.3	224	2.8	8	425	1	12.9	1.83
2	3	735	8.38	0.37	9.01	5.7	167	1.24	8	744	2	15.2	1.41
3	1	120	8.3	0.54	4.61	2.2	12	0.44	7	512	1	5.7	1.81
4	5	673	5.06	0.52	8.26	3.9	138	1.28	6	484	2	16.6	1.78
5	1	332	4.24	0.61	9.05	2.9	142	1.19	5	397	1	11.1	1.62
6	3	357	3.42	0.16	10.27	6.9	129	1.2	18	462	1	12.4	1.34
7	5	268	2.97	0.27	9.88	4.2	24	1.36	7	399	3	4.3	1.65
8	1	387	2.38	0.36	12.49	2.7	114	1.57	13	542	2	11.2	1.5
9	3	649	8.51	0.23	13.06	7.9	241	0.94	6	446	1	13.2	2.01
10	5	579	5.02	0.31	10.03	2.6	163	2.76	20	621	3	7.7	1.19
11	1	294	7.9	0.23	12.42	3.7	15	1.75	14	540	1	7.3	0.94
12	0	869	6.86	0.54	11.53	6.9	142	2.79	11	647	2	13.8	1.19
13	1	883	5.11	0.33	10.02	5.8	137	2.99	9	512	3	15.8	1.44
14	1	467	8	0.48	13.1	3.9	130	0.84	18	561	3	18.8	1.4
15	0	254	8.97	0.22	8.23	1.6	23	3.92	14	543	1	6.8	0.94
16	0	925	7.1	0.21	7.01	7.9	199	0.79	9	442	1	15.8	2.02
17	0	223	5.91	0.62	2.01	1.8	33	0.62	6	400	3	3.9	1.62
18	1	352	4.74	0.47	9.51	4.2	98	2	7	460	2	13.8	1.1
19	1	674	4.31	0.33	13.61	2	163	1.88	15	622	2	14.8	1.21
20	0	146	5.19	0.35	5.21	1	142	0.74	8	750	1	7.9	1.4

*U*_11_ represents the type of damage, *U*_12_ represents the number of acoustic emission events, *U*_13_ represents the maximum amount of drilling debris, *U*_14_ represents the solidity coefficient, *U*_21_ represents gas content, *U*_22_ represents gas emission, *U*_23_ represents electromagnetic radiation intensity, *U*_24_ represents gas pressure, *U*_25_ represents the initial velocity of gas emission, *U*_31_ represents vertical depth, *U*_32_ represents geological structure, *U*_33_ represents ground stress, and *U*_34_ represents the thickness of soft layered coal body.

According to Eqs. (7) to (9), the objective weights of each indicator are calculated as follows: *W*_*1*_ = (0.343,0.240,0.186,0.231); *W*_*2*_ = (0.256, 0.097 ,0.189, 0.198,0.260); *W*_*3*_ = (0.247,0.388,0.194,0.171).

According to Eq. (10), the combined weights of IAHP and improved CRITIC method are: *Q* = (0.054,0.058,0.045,0.068,0.048,0.069,0.049,0.060,0.088,0.134,0.202,0.082,0.043).

**Figure 4:**
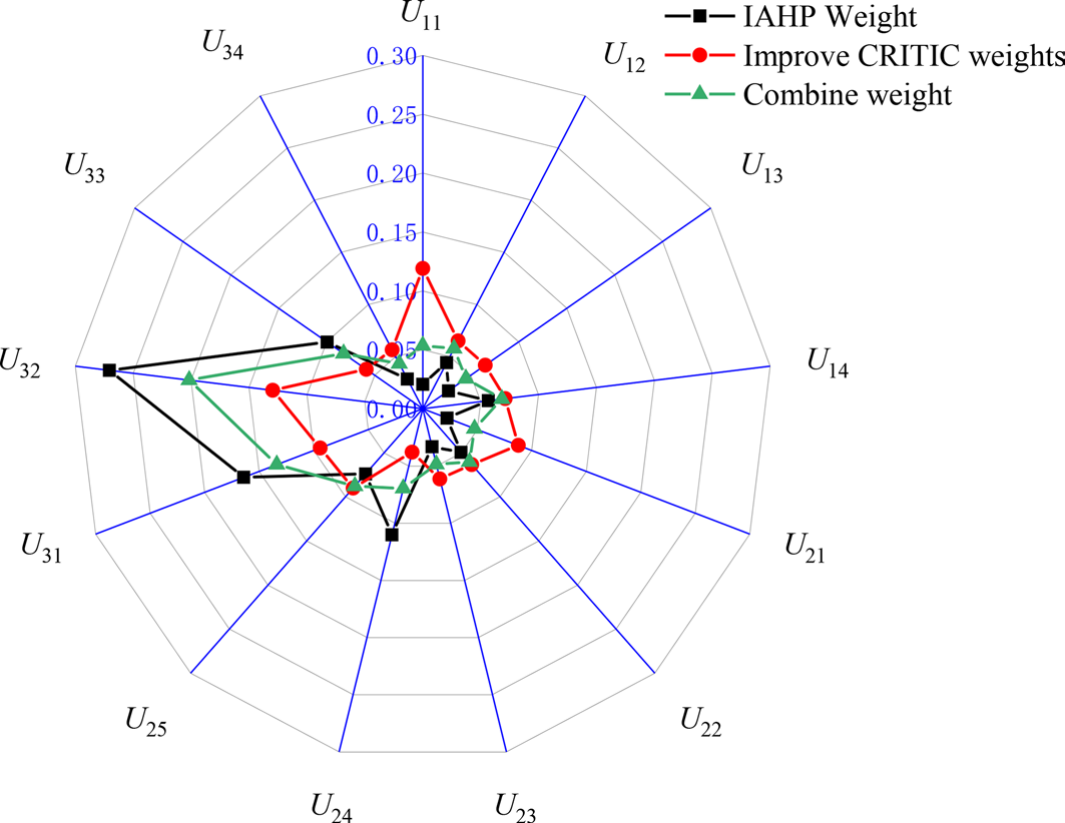
Indicator weight radar chart.

The subjective weights derived from the comprehensive IAHP method, the objective weights obtained through the improved CRITIC method, and the optimal combination weights resulting from Eq. (11) are presented in Table 4 and Fig. 4.

**Table 4:** Optimal combination weight value.

Primary index	Weight	Secondary index	IAHP method weight	Improve CRITIC method weight	Combined weight
*U*_1_	0.148	*U*_11_	0.141	0.343	0.054
		*U*_12_	0.297	0.240	0.058
		*U*_13_	0.180	0.186	0.045
		*U*_14_	0.382	0.231	0.068
*U*_2_	0.289	*U*_21_	0.077	0.256	0.048
		*U*_22_	0.171	0.198	0.060
		*U*_23_	0.115	0.189	0.049
		*U*_24_	0.381	0.097	0.069
		*U*_25_	0.256	0.260	0.088
*U*_3_	0.563	*U*_31_	0.292	0.247	0.134
		*U*_32_	0.481	0.388	0.202
		*U*_33_	0.176	0.194	0.082
		*U*_34_	0.051	0.171	0.043

#### Construction of a comprehensive assessment cloud

Hire industry experts to rate the risk of coal and gas outburst, and calculate the cloud model parameters for each evaluation index according to Eq. (14), as shown in Table 5.

**Table 5:** Cloud modeling parameters.

Evaluating indicator	Cloud model parameters
*U*_11_	(67.7,2.63,0.833)
*U*_12_	(68.1,2.38,0.122)
*U*_13_	(68.6,2.46,0.482)
*U*_14_	(69.2,2.51,0.572)
*U*_21_	(67.6,2.91,0.692)
*U*_22_	(67.9,2.41,0.295)
*U*_23_	(66.7,3.21,0.711)
*U*_24_	(67.3,1.88,0.836)
*U*_25_	(67.2,3.76,1.896)
*U*_31_	(69.0,2.76,0.519)
*U*_32_	(61.2,3.13,0.531)
*U*_33_	(65.5,2.13,1.137)
*U*_34_	(68.2,1.50,0.602)

### Evaluation and analysis

**Figure 5:**
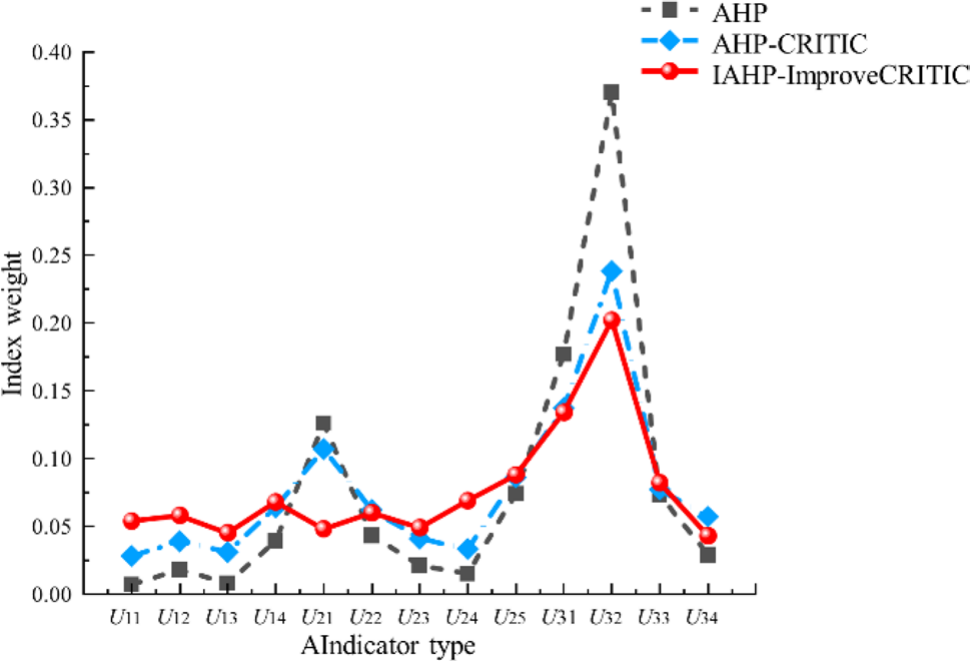
Comparison of weight values of different methods.

In order to further emphasize the feasibility of the improved combination weighting method proposed in this article, a comparison was made between the traditional AHP method and the combination weighting method of traditional AHP and CRITIC for secondary indicator weights, as presented in Table 6 and Fig. 5. From Table 6 and Fig. 5, it is evident that the AHP method is excessively subjective and overlooks objective factors related to indicator values. The weight obtained from the traditional CRITIC method shows positive correlation with volatility and conflict within indicator data. On the other hand, by combining IAHP with an improved CRITIC approach, our proposed combination weighting method incorporates both subjective and objective factors while fully considering inherent attributes of data itself as well as evaluation purposes, resulting in a more reasonable weight distribution. Furthermore, through comparative analysis of indicator weights, it becomes apparent that after integrating IAHP with the improved CRITIC approach, there is a relatively uniform distribution of combined weights. This indicates that utilizing this combined weighting methodology can effectively mitigate influences from both subjective and objective factors on individual indicator weights.

**Table 6:** Weight results obtained by different methods.

Method	*U*_11_	*U*_12_	*U*_13_	*U*_14_	*U*_21_	*U*_22_	*U*_23_	*U*_24_	*U*_25_	*U*_31_	*U*_32_	*U*_33_	*U*_34_
AHP	0.007	0.018	0.008	0.039	0.126	0.043	0.021	0.015	0.074	0.177	0.37	0.073	0.029
AHP-CRITIC	0.028	0.039	0.031	0.064	0.107	0.062	0.041	0.033	0.086	0.137	0.238	0.077	0.057
IAHP- Improve CRITIC	0.054	0.058	0.045	0.068	0.048	0.06	0.049	0.069	0.088	0.134	0.202	0.082	0.043

The digital features of clouds (*E*_*x*_, *E*_*n*_, *H*_*e*_) reflect the overall characteristics of evaluation. The distribution expectation *E*_*x*_ represents the quantification point of qualitative concept, which corresponds to the center of gravity position of the cloud droplet group in the domain. A higher value of *E*_*x*_ indicates a higher comprehensive evaluation for the sample. Entropy *E*_*n*_ is used to comprehensively measure the fuzziness and probability of qualitative concepts, representing the uncertainty and fuzziness in cloud droplet distribution. A larger value of *E*_*n*_ indicates a wider distribution span on the cloud map and a more unstable comprehensive evaluation. Super entropy *H*_*e*_ measures uncertainty in entropy and reflects condensation degree of cloud droplets. As *H*_*e*_ increases, condensation degree decreases and evaluators have greater uncertainty in their evaluations, indicating a lower level of identification with the evaluation.

**Figure 6:**
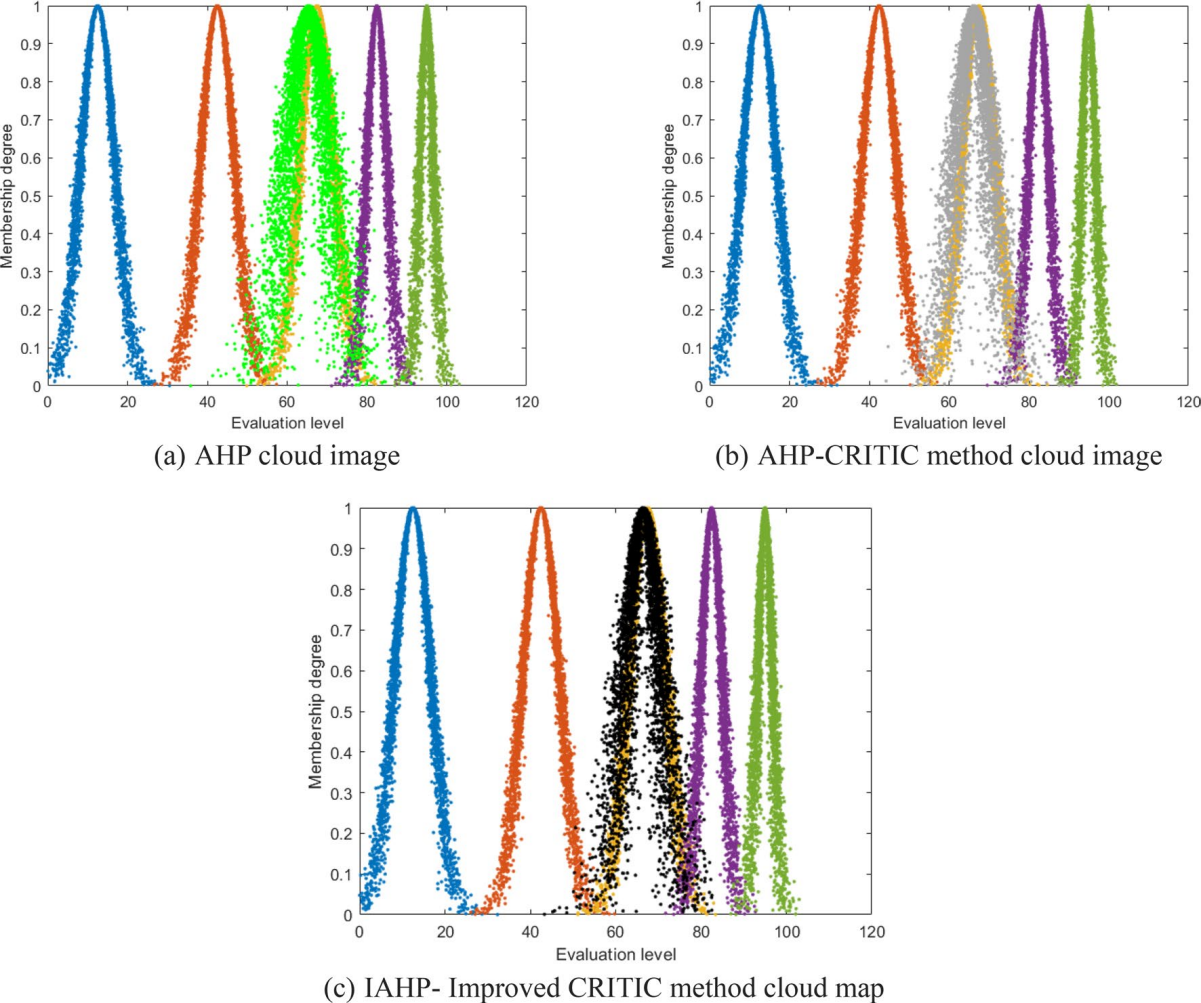
Comprehensive evaluation cloud map of different methods.

To visually demonstrate the feasibility of the IAHP improved CRITIC combination weighting method, Eq. (15) was utilized to calculate the weights of indicators and cloud model parameters for each indicator, thereby obtaining digital features of the cloud model. The specific values are presented in Table 7. Simultaneously, Matlab2023a software was employed to generate standard cloud maps at different evaluation levels based on the digital features obtained from three methods for coal and gas outburst risk assessment. By superimposing the standard cloud map with the comprehensive evaluation cloud, a comprehensive evaluation cloud map was derived as depicted in Fig. 6.

**Table 7:** Numerical characteristics of cloud models with different methods.

Method	The digital characteristics of cloud model were evaluated comprehensively
AHP	(65.38, 5.03, 1.99)
AHP-CRITIC	(66.23, 4.33, 1.65)
IAHP- Improve CRITIC	(66.46, 4.10, 1.51)

Based on Table 7 and Fig. 6, it is evident that the IAHP improved CRITIC combination weighting method proposed in this article yields a relative error of 1.54% between the distribution expectation *E*_*x*_ and the standard cloud in the comprehensive evaluation of coal and gas outburst risk. In contrast, the AHP-CRITIC combination weighting method results in a relative error of 1.89%, while using only AHP leads to a relative error of 3.14%; The relative error between the entropy *E*_*n*_ obtained using the IAHP improved CRITIC combination weighting method and the standard cloud's entropy is 2.38%. The relative error of the entropy obtained using the AHP-CRITIC combination weighting method is 3.1%, while that obtained using the AHP method has a relative error of 19.8%. Additionally, the hyperentropy *H*_*e*_ acquired through the IAHP improved CRITIC combination weighting method closely approximates the hyperentropy of the standard cloud with minimal dispersion. Therefore, the IAHP improved CRITIC combination weighting method proposed in this article exhibits a relatively concentrated distribution of cloud droplets in the comprehensive evaluation map of coal and gas outburst risk, displaying minimal fluctuations. The obtained results demonstrate high reliability, with the majority of cloud droplets falling within the "III" evaluation category and closely overlapping with it, while only a few are distributed in the "IV" region. Based on the principle of maximum membership degree, we can conclude that the level of danger associated with coal and gas outbursts is classified as "III," indicating an average level of risk. Consequently, it is imperative to enhance measures for preventing outburst accidents and adhere strictly to production safety standards. Moreover, these risk assessment findings align closely with the actual occurrence of coal and gas outbursts in this mine, thereby establishing their credibility.

## Conclusion

1. The combination weights obtained through combination weighting using the IAHP method and the improved CRITIC method can effectively reduce the impact of subjectivity and objectivity on the weights of various indicators. From the combined weights, it follows that. The solidity coefficient (*u*_14_), initial gas release velocity (*u*_25_), vertical depth (*u*_31_), geological structure (*u*_32_), and geostress (*u*_33_) have a significant impact on coal and gas outburst, and their impact on the risk of coal and gas outburst shows a relationship of *u*_32_ > *u*_31_ > *u*_25_ > *u*_33_ > *u*_14_, indicating that geological factors have a significant impact on coal and gas outburst.
2. According to the improved combination weighting cloud model, the risk level of coal and gas outburst is Level III, and the comprehensive evaluation shows that the cloud droplets are concentrated without significant fluctuations. The reliability of the calculated results is high, indicating that there is a certain risk of coal and gas outbursts during the production process of the mine. In real production, it is necessary to strengthen mine gas monitoring and emergency training and management to ensure the maximum reduction of losses from explosion accidents.
3. The weight results and digital features obtained by the IAHP improved CRITIC combination weighting method proposed in this article were compared and analyzed with those of the traditional AHP method and AHP-CRITIC combination weighting method. The results demonstrated that, compared to other methods, the relative error of the expected distribution decreased by 1.6% and 0.35%, respectively, for the IAHP improved CRITIC combination weighting method; similarly, the relative error of entropy value decreased by 17.42% and 0.72%, respectively. Furthermore, among these three methods, it was observed that the proposed IAHP improved CRITIC combination weighting method exhibited the smallest superentropy value, indicating its superior accuracy, effectiveness, and strong engineering applicability.

## Data Availability

All data generated or analysed during this study are included in this published article and its supplementary information files.
